# The application of close incisional negative pressure wound therapy in revision arthroplasty among asian patients: a comparative study

**DOI:** 10.1186/s42836-021-00094-4

**Published:** 2021-11-03

**Authors:** Ping Keung Chan, Wing Chiu Fung, Kar Hei Lam, Winnie Chan, Vincent Wai Kwan Chan, Henry Fu, Amy Cheung, Man Hong Cheung, Chun Hoi Yan, Kwong Yuen Chiu

**Affiliations:** 1 Department of Orthopaedics and Traumatology, Queen Mary Hospital, Hong Kong SAR, China; 2 Department of Orthopaedics and Traumatology, The University of Hong Kong, Hong Kong SAR, China; 3 Department of Nursing, Queen Mary Hospital, Operation Theatre Services, Hong Kong SAR, China

**Keywords:** Closed-incisional negative pressure wound therapy, Surgical site infection, Periprosthetic joint infection, Arthroplasty

## Abstract

**Introduction:**

Peri-prosthetic joint infection (PJI) was one of the main causes of revision of arthroplasty. In order to reduce wound complications and surgical site infections, close incisional negative pressure wound therapy (ciNPWT) has been introduced into arthroplasty. This study was designed to review the clinical benefits of the application of ciNPWT in revision arthroplasty.

**Methods:**

This was a single-centre retrospective comparative study approved by the Institutional Review Board. Patients, who underwent revision total knee arthroplasty or revision total hip arthroplasty at the author’s institution from January 2016 to October 2019, were included in this study. The ciNPWT cohort included all eligible patients, who underwent operations from January 2018 to October 2019, with the use of ciNPWT(*n* = 36). The control cohort included all eligible patients, who underwent operations from January 2016 to December 2017 with the use of conventional dressing(*n* = 48). The incidences of wound complications were compared to both cohorts.

**Results:**

There was a statistically significant difference in the rate of superficial surgical site infection (SSI) between control cohort and ciNPWT cohort (12.5% in control vs 0% in ciNPWT, *p* = 0.035). However, there was no statistically significance of the overall wound complication rate for both cohorts. (14.6% in control vs 8.3% in ciNPWT, *p* = 0.504).

**Conclusions:**

The application of ciNPWT could result in a lower rate of superficial surgical site infection when compared with conventional dressing among the patients undergoing revision total knee and total hip arthroplasties.

**Trial registration:**

UW19-706

## Introduction

Complications to wound healing can be detrimental to the clinical outcomes after arthroplasty. It can lead to major complications including infection and need for subsequent surgery. Among patients having primary total knee arthroplasties, 0.33% had early wound complications, which subsequently resulted in further surgical management within 30 days [1]. Those patients requiring surgery for wound complications from the first 30 days had 7–13 times higher risk of deep infection when compared to those without wound problems [1, 2]. Measures to prevent wound complications were even more important to revision arthroplasty as revision total knee arthroplasty was associated with a 15 fold increase in postoperative complications rates relative to primary knee arthroplasty [3].

Various measures were advocated to optimize wound healing after arthroplasty. These included preoperative optimization of modifiable risk factors such as nutritional status, smoking status, and diabetic control. Intraoperatively, surgeon should have meticulous handling of the skin and soft tissue. Advanced dressing management is another important area, which has recently got more attention as more scientific evidence proven it clinical effectiveness in arthroplasty.

Traditionally, cotton gauze fabric was widely used in wound care because of its excellent absorption power and low cost. However, other advanced dressing materials were recently showed to have more meaningful clinical benefits. The application of a commercial silver-impregnated occlusive dressing, which is a hydrofiber dressing with antibi-microbial ionic silver, was showed to result in a fourfold reduction in acute periprosthetic joint infection when compared with conventional sterile gauze dressing [4]. Closed-incisional negative pressure wound therapy (ciNPWT) is another evidence-based option, which was put in the place of field of arthroplasty for over 10 years [5]. A recent randomized controlled trial comparing the effectiveness of ciNPWT versus silver-impregnated dressing showed that ciNPWT was statistically significant more effective in reducing the 90-day postoperative surgical site complication (ciNPWT: 3.4% vs silver-dressing: 14.3%; odds ratio (OR): 0.22; *p* = 0.0013) [6].

The proposed primary mechanisms of ciNPWT in promoting wound healing include: (i) macrodeformation by drawing wound edges together, (ii) microdeformation by facilitating cell proliferation, and (iii) fluid removal from extra-cellar matrix and environmental control to keep the wound insulated, warm and hydrated [7]. It has been propose that ciNPWT could better protect incisions against external contamination compared to conventional dressing [8]. Studies were also proposed that ciNPWT facilitates healing by improving perfusion, reducing oedema and preventing the formation of haematoma [9, 10]. Long-term benefits include improving the incision quality in terms of mechanical strength and histological properties [11–14].

The cost of the application of these advanced dressing materials is still a concern. The cost of single-use, disposable reported in the literature was between $500 and$600 USD [15]. In Asia, limited literatures were expected to be released to discuss the application of ciNPWT in arthroplasty. One of the reasons of low utilization for ciNPWT in Asia might be due to the cost consideration.

Revision arthroplasty was deemed to be a major risk factor of wound related complications. According to the national registries from England and Finland, revision arthroplasty showed an increased risk of postoperative infection-related complications when compared to primary arthroplasty [16, 17]. A recent review studying the clinical effectiveness of ciNPWT in arthroplasty showed that ciNPWT was indicated to be used in revision arthroplasty instead of primary arthroplasty because of the relatively higher risk of wound complication in revision arthroplasty [18]. Moreover, the use of ciNPWT for infection prevention following revision total knee arthroplasty was demostrated to be cost-effective [19]. Based upon the cost-effectiveness consideration, ciNPWT was mainly utilized in patients having the high-risk of wound complications (revision THA and revision TKA) since January 2018 in our institution.

This study aimed to review the efficacy of the application of ciNPWT in revision arthroplasty when compared with conventional dressing in the authors’ institution, which is one of the major joint replacement centres in Asia.

## Methods

### Study design

This was a single-centre, retrospective comparative study approved by the Institutional Review Board (HKU/HA HKW IRB) (Reference no. UW19-706) in a tertiary referral university hospital.

**Fig. 1:**
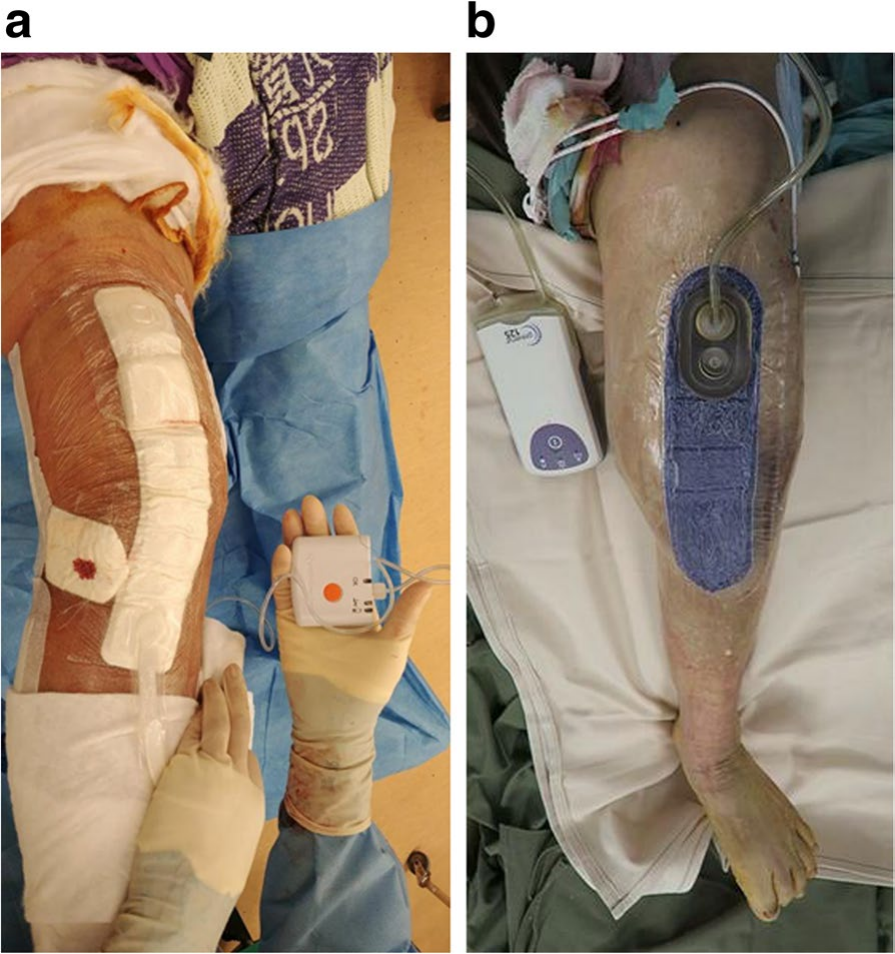
ciNPWT devices used in revision arthroplasties. **a** PICO system **b** Prevena system

Patients, who underwent revision TKA or THA at our institution from January 2016 to October 2019, were included in this study. The conventional dressing used traditionally was Cosmopor® E (Hartmann), which is a sterile, adhesive wound dressing made of soft, non-woven polyester. Since January 2018, the surgical team changed the dressing to ciNPWT, which were either Prevena Incision Management System (Acelity, San Antonio, TX) or PICO (Single use ciNPWT system, Smith & Nephew, Hull, UK) (Fig. 1). The change of practice in wound dressing provided us with 2 naturally formed cohort groups: those patients who underwent surgery between January 2016 to December 2017 received a conventional dressing, whereas those patients who underwent surgery between Jan 2018 to October 2019 received ciNPWT. All patients were treated by the same surgical team consisting of four fellowship-trained surgeons, who performed revision surgery with similar surgical techniques.

The patients in both cohorts were managed according to the same perioperative protocols with the difference mainly in the dressing material used at the end of the surgical procedures. Prophylactic antibiotics, single dose of 1 g cefazolin, were given 30-min before the surgical incision. For revision surgery due to aseptic causes e.g. aseptic loosening or periprosthetic fracture, cefazolin would only be used for 24-h after the operation. For revision surgery because of infection, 2-stage revision surgery would be performed. The perioperative antibiotic used was according to the recommendation by clinical microbiologist based on the antibiotic sensitivity profile of the bacteria. To decrease perioperative blood loss, Intravenous tranexamic acid was given at a dosage of 15 mg/kg before induction of anaesthesia, and another intravenous injection of the same dosage would be given 4-h afterwards. For revision TKA, tourniquet was used throughout the surgical invasive procedures. Antibiotic-loaded cement (PALACOS® + G, Heraeus), was used for all cemented prosthesis. Allogenic blood transfusion was prescribed if postoperative haemoglobin drop to ≤ 7 g/dL in hemodynamically stable adults, or ≤ 8 g/dL in patients who had underly cardiovascular or respiratory comorbidities. No surgical drainage was used. All wounds were closed in layers with barbed sutures (Stratafix, Depuy Synthes).

For the postoperative wound surveillance, the dressing in the ciNPWT cohort would be kept intact for 7 days, and then changed to conventional dressing, whereas the wound condition in the conventional dressing cohort was reviewed at postoperative day 3, and then a new piece of conventional dressing would be re-applied to the surgical wound. The conventional dressing used in both cohorts would be kept intact and removed on postoperative day 14.

All the patient’s medical records and perioperative parameters were reviewed. Patient demographics, the type of revision arthroplasty, indications for surgery, mode of anaesthesia, American Society of Anaesthesiologist (ASA) grade and operative duration were recorded. Patients’ preoperative status were also documented, including haemoglobin and albumin level, comorbidities and risk factors for wound complications. Clinical photos were taken at the surgical sites with a high-resolution digital camera, and the wound conditions were documented in the wound surveillance chart by a nursing specialist, who specialized in managing surgical wounds in the orthopaedic department. The nurse was in charge of the documentation in the wound surveillance chart, documentation of the postoperative surgical wound conditions, including both septic and aseptic wound complications, during the course of recovery in the in-patient and out-patient periods. Septic complications, such as surgical site infections (SSI) and prosthetic joint infection (PJI) were recorded. SSI, including both superficial incisional SSI and deep incisional SSI, were defined according to the criteria by Centers for Disease Control (CDC) [20], while prosthetic joint infection (PJI) was defined according to the criteria provided by Musculoskeletal Infection Society (MSIS) [21]. Aseptic wound complications, such as persistent drainage, haematoma formation, wound dehiscence, suture granuloma, blister formation, and maceration of the wound, were also documented. The patient would be arranged to have wound surveillance in the out-patient follow-up at 2-week, 6-week, 3-month, 6-month and yearly after discharge from hospital. Data, including wound complications, re-operations due to wound complications of other causes, readmission within 30 days of surgery, periprosthetic joint infection, 90-day perioperative and mortality, were retrieved from the medical record system at our institution.

The primary outcomes were (1) the overall incidence of septic and aseptic wound complications, and (2) the incidence of septic and (3) the incidence of aseptic wound complications. The secondary outcomes include the length of hospital stay, re-operations due to wound complications, 90-day postoperative mortality, and readmission within 30 days of surgery. All the outcome measures were reviewed by two orthopaedic specialists individually, and the cases would be reviewed together so as to make a consensus in case a discrepancy in the individual assessments.

### Statistical analysis

The statistical analysis was performed using IBM SPSS 26.0 software (IBM, Armonk, NY, USA), and the statistically significance took place at the 5% significance level. The primary and secondary outcome measures was compared. To control for confounding factors, the baseline data between the two cohorts, including patient demographics, existing comorbidities, types of revision arthroplasty performed, indications for revision arthroplasty, ASA grade, mode of anaesthesia, operation duration, as well as preoperative and postoperative levels of albumin and haemoglobin, were also compared.

The choice of statistical tests based on the data distribution, and the nature of the data (nominal, ordinal or interval/ratio). The independent samples t test was used for parametric data, while the chi-square or Fisher exact test was used only non-parametric data and categorical data depending on the observed frequency. All available data were incorporated in data listings and tabulations. No imputation of values for missing data were performed.

## Results

A total of 84 patients were reflected in the present study. They underwent either revision hip arthroplasty (*n* = 38) or revision knee arthroplasty (*n* = 46). The majority of cases were revised because of infection (*n* = 43, 51.2%), whereas the others were revised because of different aseptic causes (*n* = 41, 48.8%), including loosening, polythene wear, instability, fracture, and dislocation. 36 patients were enrolled in the ciNPWT cohort and 48 patients in the control cohort. Among the ciNPWT cohort, the PICO and Prevena systems were utilized or 22 and 14 patients respectively.

There were no significant differences in terms of age, sex, types of revision procedure performed, indications for revision arthroplasty, ASA grade, mode of anaesthesia, operation duration, as well as preoperative and postoperative levels of albumin and haemoglobin, between the two cohorts (Table 1). While revision arthroplasty itself being a major risk factor for wound complications, some patients had additional risk factors, most commonly history of prior joint infection (ciNPWT 63.9% Vs Control 41.7%), ASA grade ≥ 3 (ciNPWT 61.2% Vs Control 60.4%), and diabetes mellitus (ciNPWT 33.3%Vs Control 20.8%) (Table 2). Apart from the risk factor of prior joint infection reaching statistically significant (ciNPWT 63.9% Vs Control 41.7%, *p* = 0.05), other risk factors in both cohorts did not show a statistically significant difference.

**Table 1:** Patients demographics and surgical details

		ciNPWT		Control		*P*-value
		Number of patients	%	Number of patients	%	
Total Number of patients		36		48		
Age at operations	Mean age	69.4		70.9		0.557
Sex	Male	20	55.6	17	35.4	0.066
	Female	16	44.4	31	64.6	
Types of revision surgery	Hip	12	33.3	26	54.2	0.058
	Knee	24	66.7	22	45.8	
Indications	Infected	23	63.9	20	41.7	0.083
	Loosening	5	13.9	17	35.4	
	PE wear/ failure	1	2.8	4	8.3	
	Instability	1	2.8	2	4.2	
	Wound drainage	0	0	2	4.2	
	Fracture	1	2.8	2	4.2	
	Dislocation	4	11	1	2.1	
	Flexion contracture	1	2.8	0	0	
ASA Grade	≤ 2	15	41.7	19	39.6	0.847
	≥ 3	21	58.3	29	60.4	
Anesthesia	General / General & regional	27	75	39	81.3	0.490
	Spinal / Combined spinal epidural	9	25	9	18.8	
Operation Duration (min)	Mean	207.2		225.5		0.376
Haemoglobin	Per-operative mean	11.5		11.9		0.322
	Post-operative mean	9.4		9.8		0.306
	Transfusion	11	30.6	11	22.9	0.431
Albumin	Pre-operative mean	38.31		39.83		0.243
	Post-operative mean	28.56		27.77		0.530

*ASA* American Society of anesthesiologis

**Table 2:** Patient risk factors / Comorbidities

	ciNPWT		Control		*P*-value
	Number of patients	%	Number of patients	%	
Total Number	36		48		
Risk Factors					
Prior Joint Infection	23	63.9	20	41.7	0.050
ASA ≥ 3	21	58.3	29	60.4	0.847
Diabetes Mellitus	12	33.3	10	20.8	0.197
Smoker	12	33.3	11	22.9	0.289
Cardiovascular Disease: IHD / Heart Failure / CABG / PCI before	10	27.8	12	25.0	0.774
Obesity (BMI ≥ 30)	9	25.0	10	20.8	0.651
Active Cancer / Previous Cancer with RT to surgical site	4	11.1	8	16.7	0.471
Pre-operative Albumin ≤ 30	4	11.1	2	4.2	0.395
Liver Disease	2	5.7	1	2.1	0.570
Renal Failure	2	5.6	2	4.2	1.000
Active Infection / Sepsis	2	5.6	3	6.3	1.000
Depression / Schizophrenia	2	5.6	1	2.1	0.574
Deep Vein Thrombosis	1	2.8	1	2.1	1.000
B12 Deficiency Anaemia	1	2.8	1	2.1	0.676
Current use of corticosteroid / Immunosuppressant	0	0	2	4.2	0.504

*BMI* Body Mass index, *RT* Radiotherapy, *IHD* Ischaemic Heart Disease, *CABG* Coronary artery bypass graft, *PCI* Percutaneous coronary intervention, *ASA* American Society of anesthesiologists

The bacteriology in PJI cases in both cohorts were reviewed in details (Table 3). There were no statistically significant differences in the septic and aseptic revision case distribution in both cohorts (septic revision: ciNPWT 63.9% Vs Control 41.7%, *p* = 0.082). For the septic revision cases, the diagnosis of PJI was based on the MSIS criteria [21]. There was also no statistically significant difference in culture negative PJI in both cohorts (ciNPWT 44% Vs Control30%, *p* = 0.362). Among PJI cases having a positive bacterial culture, there was no statistically significant difference in the bacteriological profile. Methicillin sensitive staphylococcus aureus (MSSA) was the most commonly found organism in both cohorts, and there were no statistically significant differences in case distribution in both cohorts (MSSA: ciNPWT 69.2% Vs Control: 78.6%, *p* = 0.454). And there were no antibiotic resistant organisms, eg methicillin resistant staphylococcus aureus, in both cohorts.

**Table 3:** Comparison of bacteriology of PJI cases in the cohorts

	ciNPWT(%)	Control(%)	*P*-value
Culture negative PJI	44%	30%	0.362
Culture positive PJI	56%	70%	0.362
Among Culture positive PJI			
• Methicillin sensitive Staphylococcus aureus	69.2%	78.6%	0.454
• Streptococcus	15.4%	0%	0.222
• Diphtheroids	7.7%	7.1%	0.741
• Other organism	7.7%	14.3%	0.529
• Antibiotic resistant organism eg MRSA, ESBL E-coli, VRSA	0%	0%	1.000
• With gram positive Bacteria ^a^	92.3%	100%	0.481
• With gram negative Bacteria ^a^	15.4%	7.1%	0.471
• MSSA	69.2%	78.6%	0.454
• Non-MSSA	30.8%	21.4%	0.454
• PJI with positive culture of 1 bacteria	92.3%	92.9%	0.741
• PJI with positive culture > = 2 bacteria	7.7%	7.1%	0.741

*MRSA* Methicillin-resistant Staphylococcus aureus, *ESBL E-coli* Extended Spectrum Beta-Lactamase Escherichia coli, *VRSA* Vancomycin-resistant Staphylococcus aureus, *MSSA* Methicillin sensitive Staphylococcus aureus

^a^Not add up to 100% because the culture results could have 2 bacteria, one gram positive and one gram negative

Across both cohorts, 10 patients (11.9%) developed wound complications, including superficial SSI (*n* = 6), persistent drainage (*n* = 3), blister formation (*n* = 1) and haematoma formation requiring drainage (*n* = 1). There was one case of reoperation due to superficial SSI, other cases of superficial SSI were treated with antibiotic only.

**Fig. 2:**
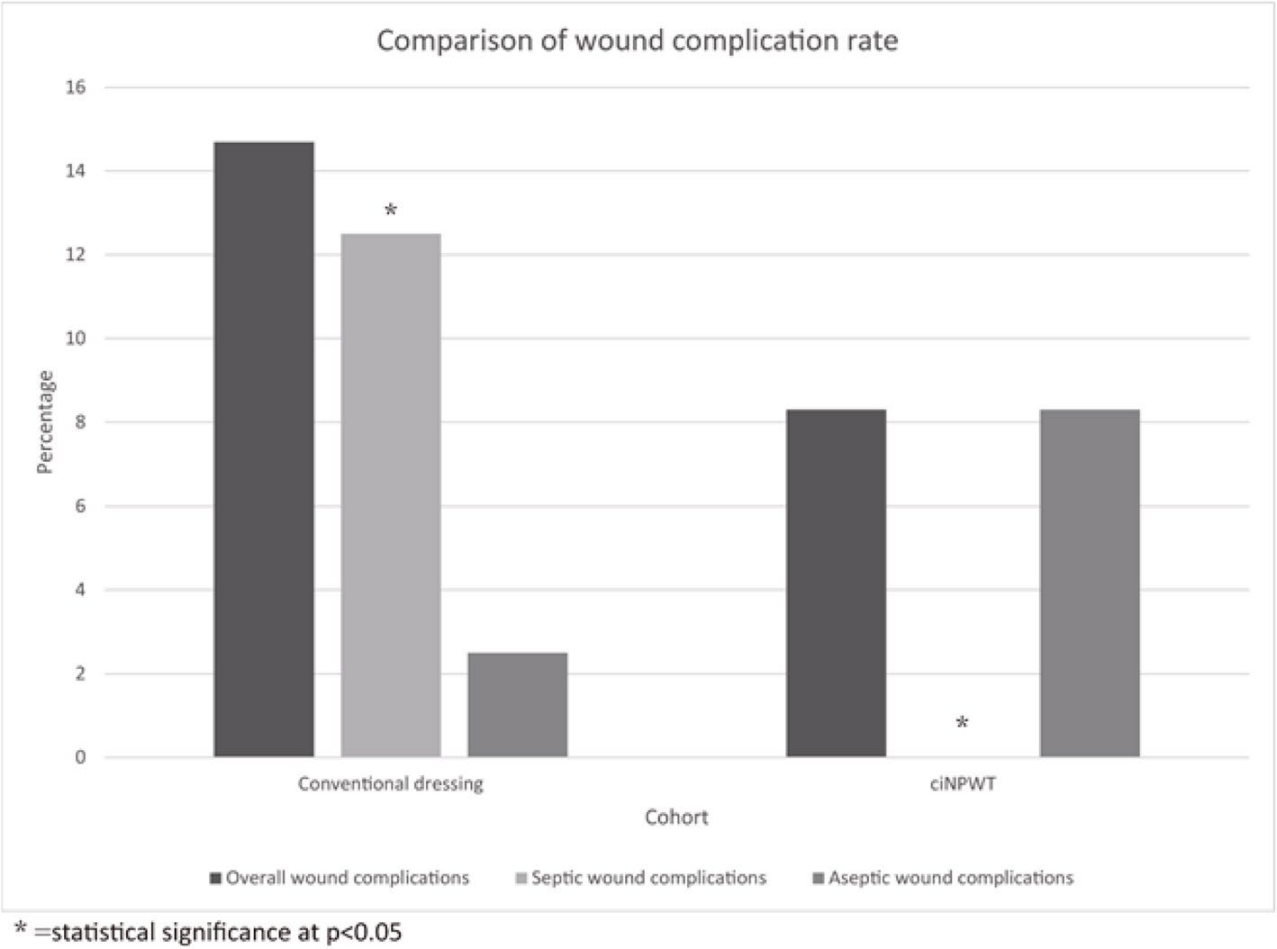
Comparison of wound complication rate. *= statistical significance at *p*<0.05

The overall wound complication rate of the ciNPWT cohort was 8.3% (*n* = 3, 2 persistent drainage, 1 blister formation) compared to 14.6% for the control cohort (*n* = 7, 6 superficial SSIs, 1 persistent drainage). Hence, the overall wound complication rates of the two cohorts did not differ statistical significantly (*p* = 0.504) (Table 4, Fig. 2). Septic wound complications (SSI and PJI) and aseptic wound complications (persistent drainage, haematoma formation, wound dehiscence, suture granuloma, blister formation, and maceration of the wound) were compared separately. The rate of septic wound complication was statistically significantly higher in the control cohort than the ciNPWT cohort (ciNPWT 0% Vs Control 12.5%, *p* = 0.035). One patient with superficial SSI subsequently developed PJI, which required 3 reoperations, and eventually succumbed due to persistent bacteremia. The rate of aseptic wound complications did not differ significantly in both cohorts (ciNPWT 8.3% Vs Control 2.5% Vs, *p* = 0.309).

**Table 4:** Summary of primary and secondary outcomes

	ciNPWT		Control		*P*-value
	Number of patients	%	Number of patients	%	
Total Number	36		48		
Primary outcomes					
Overall wound complications	3	8.3	7	14.6	0.504
Septic wound complications	0	0	6	12.5	0.035*
Aseptic wound complications	3	8.3	1	2.5	0.309
Secondary outcomes					
Length of hospital stay (Mean)	31.5		22.9		0.125
Re-operations due to wound complications	0	0	1	2.1	1.000
90-day perioperative mortality	0	0	1	2.1	1.000
Readmission within 30 days of surgery	0	0	0	0	1.000

^*^ = statistical significance at *p* < 0.05

There were no statistically significant differences in the secondary outcomes which included the length of hospital stay (ciNPWT 31.5 ± 28.6 Vs Control 22.9 ± 19.2,, *p* = 0.125), re-operations due to wound complications (ciNPWT 0% Vs Control: 2.1%,, *p* = 1.000), 90-day perioperative mortality (ciNPWT 0% Vs Control: 2.1%,, *p* = 1.000) and readmission within 30 days of surgery (ciNPWT 0% Vs Control: 0%,, *p* = 1.000).

## Discussion

The basic scientific mechanism, and the advantages of ciNPWT were well-reported [22–24]. Immediate effects of ciNPWT include protecting the incision from external contamination, decreasing the lateral tension applied on the incision, increasing the appositional strength, normalizing stress distribution and increasing skin perfusion. Long-term benefits include improving incision quality, in terms of the mechanical strength, histological and gene expression profile.

A recent meta-analysis of 11 studies on ciNPWT in knee or hip arthroplasty (including 8 randomized trials and 3 comparative cohort studies) found that ciNPWT could significantly reduce the incidence of wound complication and SSI in high-risk patients, and patients undergoing revision arthroplasties when compared with conventional dressing [25]. Among the high-risk patients, the ciNPWT cohort had significantly lower rates of wound complication (OR = 0.38, *p* = 0.030) and SSI (OR = 0.24, *p* = 0.005). Likewise, among patients undergoing revision arthroplasty, the ciNPWT cohort had lower rates of wound complication (OR = 0.33, *p* < 0.001) and SSI (OR = 0.26, *p* = 0.004). However, there were no significant differences in wound complication and SSI between ciNPWT versus conventional dressing among non-high-risk patients and patients undergoing primary arthroplasty. Another meta-analysis involving 8 randomized trials reported a significantly lower overall SSI risk for primary and revision arthroplasty in patients having ciNPWT compared to conventional dressings, and specifically lower in revision THA and TKA [26]. However, ciNPWT may increase the risk of noninfectious complications after primary TKA, such as blisters, seroma, hematoma, persistent drainage and wound dehiscence [26].

Existing studies demonstrated the effectiveness of ciNPWT in decreasing wound complications, specifically in revision arthroplasty. In a 2016 comparative study involving high-risk patients with multiple risk factors for SSIs, the ciNPWT group had significantly fewer wound complications (6.7% vs 26.9%, *p* = 0.024) and SSIs (3.3% vs 18.5%, *p* = 0.045) compared to patients treated with antimicrobial dressings [27]. In a 2019 randomised controlled cohort study involving revision arthroplasty in patients with risk factors for wound complications [28], the ciNPWT group had a significantly lower wound complication rate (10.1% vs 23.8%, *p* = 0.022) and rate of re-operation (2.5% vs 12.5%, *p* = 0.017) than the control group (who received conventional dressing). However, ciNPWT was not found to have any significant effect on reducing the number of superficial or deep surgical site infections.

The majority of the literatures studying the use of ciNPWT in arthroplasty were from North America. This might be explained by the current marketing strategies by the industrial companies in targeting the users of ciNPWT devices in the North America. At present, the ciNPWT market was primarily located in North America, with a revenue of USD 738.1 million in 2018 [29]. Nevertheless, the demand for ciNPWT was projected to increase in the Asia–Pacific region, especially In China. The ciNPWT market was expected to grow at a tremendous high rate between 2020 and 2027 in China as the growing economy expediate the market demand for a better dressing material for the postoperative surgical wound management [30].

There is a paucity of clinical data from local and Asian regions. In a recent comparative study in China involving patients undergoing total ankle replacement, the ciNPWT group had lower rates of wound complication 7.7% vs 19.0%, *p* = 0.34) and infection (0% vs 4.8%, *p* = 0.62, [31], although these differences were not statistically significant. Hence, our study aimed to further investigate the role of ciNPWT in Asian patients undergoing arthroplasty. Our study showed that ciNPWT could reduce the rate of superficial surgical site infections (0% vs 12.5%, *p* = 0.035) when compared to conventional dressing. Because of the convincing data showed in our study, ciNPWT is currently used for all patients who undergo revision arthroplasty in our institution.

**Fig. 3:**
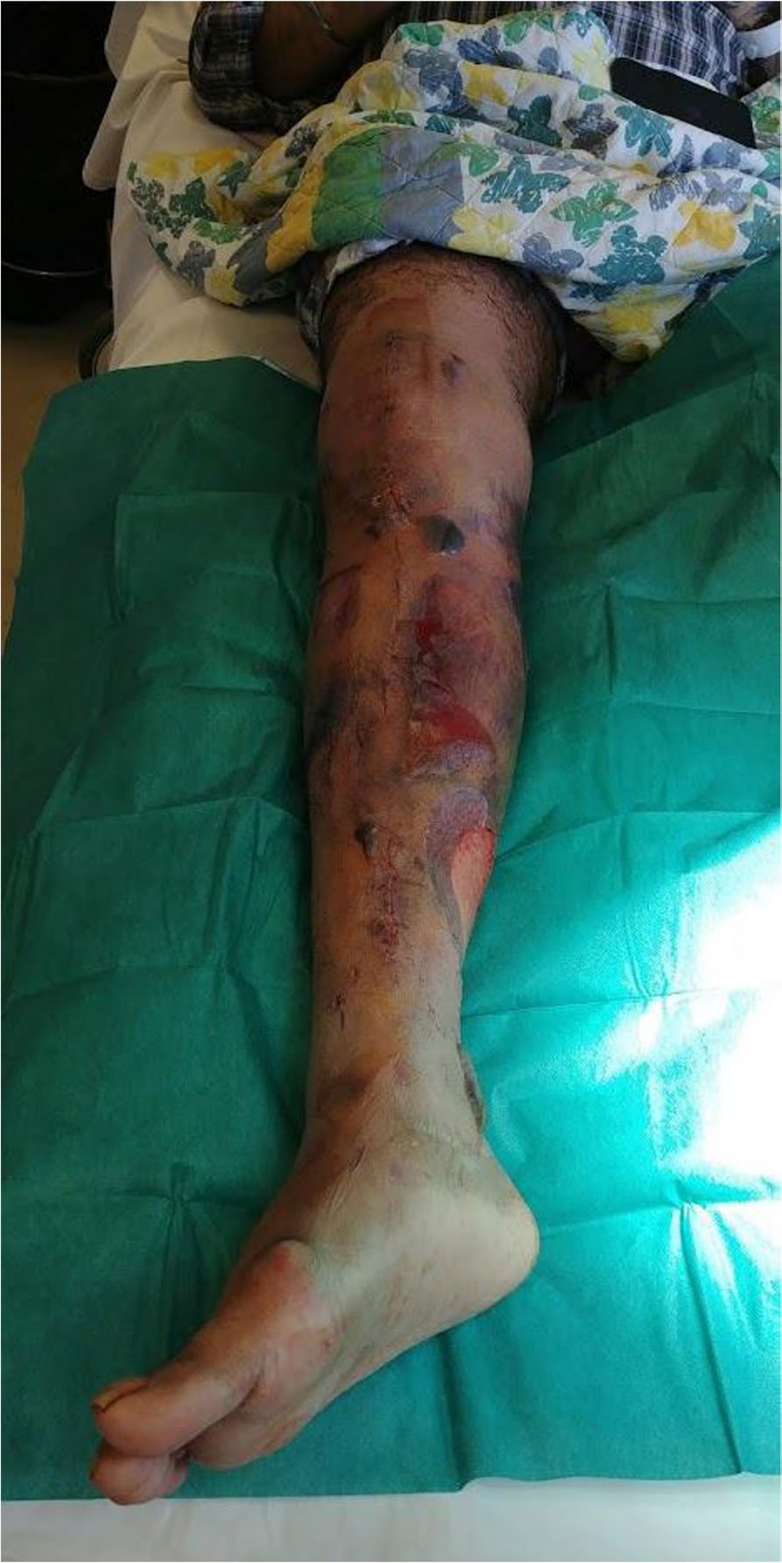
Blisters were observed medial to surgical incision, which subsequently resolved with conventional dressing

In our study, it was interesting to note that the overall wound complication rate of the ciNPWT cohort and control cohort did not differ significantly (8.3% vs 14.6%, *p* = 0.504). It was caused by the higher number of non-infectious wound complications in ciNPWT cohort, in particular blister formation. The increase in the blister complication in the surgical wound among ciNPWT cohort might result from inadequate experience in the application of ciNPWT during the initial use. Although the difference was not statistically significant, the precautions in the application of ciNPWT to avoid blister formation should be taken. An earlier study using ciNPWT in total knee arthroplasty was prematurely terminated due to blister formation and maceration of the surrounding skin [32]. Vaez-zadeh proposed blisters could form at the interface of the foam edge and the transparent film under the effect of negative pressure, if the skin is not well protected by transparent film in accordance with the manufacturer’s recommendations [33]. In our study, a patient was complicated by the blister formation in the ciNPWT cohort on postoperative day 4 (Fig. 3). ciNPWT was stopped, and the conventional dressing was applied. Blisters resolved eventfully without any further complications. To avoid blister formation, the following procedures were recommended during the application of ciNPWT. First, the skin around the surgical wound should be cleaned up and dried thoroughly to ensure no foreign products interfering the negative suction. Second, the foam dressing should be accurately positioned to avoid the foam edge placing directly over edges of the surgical wound because the negative suction force may cause secondary injury to the wound edges. Third, it is important to ensure that the transparent film applied over the foam does not create excessive air spaces or bubbles around the foam dressing, which may affect the pressure distribution on the intact skin around the foam dressing. When the transparent film was applied to the knee, the knee should be flexed around 60 degrees so as to minimize the maceration of the skin upon knee flexion. Fourth, daily checking and documentation of the peri-wound skin condition should be undertaken by the medical and nursing team.

One of the concerns of using ciNPWT was the cost of the device, but it was showed to be cost-effective paradoxically in further studies. The main reasons for hospital readmission after hospital discharge among patients having arthroplasty were infection and wound complication, which contributed 35.9% and 14.4% of the unplanned readmissions respectively [34]. By reducing wound complications, ciNPWT could potentially result in cost savings by reducing hospital readmission, and the subsequent surgical management such as reoperation and debridement. In a UK cost-effectiveness analysis on primary hip and knee arthroplasty, an estimated USD1607 per patient could be saved by using a single-use ciNPWT device instead of conventional dressings, with greater savings demonstrated in high-risk subgroups such as patients with obesity and ASA grade ≥ 3 [35].

There are limitations to this study. First, two models of ciNPWT device, including PICO and Prevena, were applied in the current study, and this may cause heterogeneity in our results. The prinicipal difference between two devices is the design in foam layers: Prevena having a single layer of foam dressing, while PICO having 4 layers of dressing. Since there was no study demonstrating any significant clinical difference between the two products, they were not analyzed separately in our study. Second, although baseline characteristics of the two cohorts were compared, it is possible that some confounders may be missed due to the retrospective nature of our study. Third, the antibiotic used postoperatively among the septic revision cases were not compared because of the heterogenicity of the antibiotic used. This might contribute to the confounding factor. Finally, hip and knee revision arthroplasties, and septic and aseptic revision was not analysed separately in both cohorts because of the insufficient number of revision cases. Additionally, sub-group analysis could be performed in future study involving higher caseloads. Nevertheless, our study was among the first study to investigate the clinical outcome of ciNPWT in Asian patients after revision arthroplasties. Together with existing literature, this work opens to the way towards the use of ciNPWT for wound management in patients undergoing revision arthroplasty or at high risk of surgical site infection.

## Conclusion

The application of ciNPWT could result in a lower rate of superficial surgical site infection when compared with conventional dressing among the patients undergoing revision total knee and total hip arthroplasties.

These results supported the use of ciNPWT in patients undergoing revision arthroplasty. Proper training and precautions should be made during the application of ciNPWT to avoid blister formation.

## Data Availability

The datasets used and/or analysed during the current study are available from the corresponding author on reasonable request.

## Abbreviations

ASA: American Society of Anaesthesiologists
CDC: Centers for Disease Control
ciNPWT: Close Incisional Negative Pressure Wound Therapy
ESBL E-coli: Extended Spectrum Beta-Lactamase Escherichia coli
HKU/HA HKW IRB: Institutional Review Board of University of Hong Kong/Hospital Authority Hong Kong West Cluster
MRSA: Methicillin-resistant Staphylococcus aureus
MSSA: Methicillin sensitive Staphylococcus aureus
MSIS: Musculoskeletal Infection Society
NICE: National Institute for Health and Care Excellence
OR: Odds Ratio
PJI: Prosthetic joint infection
RR: Risk Ratio
SSI: Superficial Surgical Site Infection
THA: Total Hip Arthroplasty
TKA: Total Knee Arthroplasty
VRSA: Vancomycin-resistant Staphylococcus aureus
